# A Critical Review of Nonlinear Damping Identification in Structural Dynamics: Methods, Applications, and Challenges

**DOI:** 10.3390/s20247303

**Published:** 2020-12-19

**Authors:** Tareq Al-hababi, Maosen Cao, Bassiouny Saleh, Nizar Faisal Alkayem, Hao Xu

**Affiliations:** 1 Department of Engineering Mechanics, Hohai University, Nanjing 210098, China; tareq.alhababi@hhu.edu.cn; 2 Jiangxi Provincial Key Laboratory of Environmental Geotechnical Engineering and Disaster Control, Jiangxi University of Science and Technology, Ganzhou 341000, China; 3 Nantong Ocean and Coastal Engineering Research Institute, Hohai University, Nantong 226000, China; 4 College of Mechanics and Materials, Hohai University, Nanjing 211100, China; bassiouny.saleh@alexu.edu.eg; 5 College of Civil and Transportation Engineering, Hohai University, Nanjing 210098, China; nizar.alkayem@yahoo.in; 6 State Key Laboratory of Structural Analysis for Industrial Equipment, Faculty of Vehicle Engineering and Mechanics, School of Aeronautics and Astronautics, Dalian University of Technology, Dalian 116024, China; xuhao@dlut.edu.cn

**Keywords:** nonlinear damping identification, nonlinear damping applications, finite element modelling, structural damage detection, dynamic features

## Abstract

In recent decades, nonlinear damping identification (NDI) in structural dynamics has attracted wide research interests and intensive studies. Different NDI strategies, from conventional to more advanced, have been developed for a variety of structural types. With apparent advantages over classical linear methods, these strategies are able to quantify the nonlinear damping characteristics, providing powerful tools for the analysis and design of complex engineering structures. Since the current trend in many applications tends to more advanced and sophisticated applications, it is of great necessity to work on developing these methods to keep pace with this progress. Moreover, NDI can provide an effective and promising tool for structural damage detection purposes, where the changes in the dynamic features of structures can be correlated with damage levels. This review paper provides an overview of NDI methods by explaining the fundamental challenges and potentials of these methods based on the available literature. Furthermore, this research offers a comprehensive survey of different applications and future research trends of NDI. For potential development and application work for nonlinear damping methods, the anticipated results and recommendations of the current paper can assist researchers and developers worldwide to find out the gaps and unsolved issues in the field of NDI.

## 1. Introduction

In recent decades, a huge number of engineering structures such as various civil structures (e.g., bridges, dams, buildings, etc.), rotating machines, aircraft, etc., have been designed and widely used in real life-services [1]. Such structures, with either simple or complex geometric or material properties, are subjected to different levels of vibrations from numerous sources, including earthquakes, wind loads, vehicle motions, imbalance of rotating machines, etc. [2]. Excessive levels of vibrations largely affect the performance, health condition, and serviceability of structures and even lead to structural instability and failures [3].

In general, structural damping is a preferable dynamic characteristic able to reduce the degree of system vibrations to an acceptable level [4]. Damping and its variation occur due to the continuous dissipation of energy [5], both internally and externally, linked with factors such as material degradation, geometrical changes, boundary conditions, etc. [6]. With complex mechanisms, structural damping is commonly classified into three types. First, the fluid damping, which originates from hydrodynamic or aerodynamic forces surrounding the structures [7]. Second, the material damping, which appears due to complex atomic-molecular interactions inside materials [8]. Third, the structural damping, resulting from Coulomb friction between parts within a structural system [9]. For damping characterization, several simplified models have been suggested; for instance, viscous damping, hysteretic damping, and Coulomb frictional damping models [10]. In real applications, equivalent viscous damping is commonly used to model the overall behavior of damped systems [11].

Most structural systems show a certain extent of nonlinearity associated with different sources [12,13]. However, neglecting the nonlinearity is acceptable in many cases for the sake of simplification of analysis [14,15]. In other cases, nonlinear behavior plays a dominant role. Nonlinearity neglection should be prevented in such cases, as it may lead to erroneous predictions of system behaviors [16]. Among the causes of system nonlinearity, nonlinear damping [17,18] is often regarded as the most influential; this complexity of which makes it challenging to perform system identification [19]. Moreover, to better accommodate the development of advanced material with strong nonlinear behavior, research on the impact of nonlinear damping is of increasing importance [20,21,22].

Some damping identification approaches, theoretical and experimental, have been developed [23]. Experimental techniques for damping estimation show advantages in accuracy and reliability. Damping identification approaches provide straightforward explanations for damping properties as compared with theoretical approaches [24]. The enhancement technique of engineering structures is carried out by measuring inputs and outputs during the experiment of real structures. This enhancement technique is made to avoid unwanted behavior of the system subject to damping effects [25].

Several hypotheses can be used for linear and nonlinear damping of low-damping systems [26,27,28]. High-damping systems usually involve structural damage [29]; however, nonlinear effects on vibrating systems cannot be neglected because of their significant impact on dynamic behaviors [30,31]. Linear damping approaches can provide precise structural numerical predictions [32,33]. Nevertheless, in numerous modern applications, the effectiveness of these methods cannot be guaranteed, especially when the structures are complex, e.g., composed of composite materials or operating in a hostile environment [34,35]. Therefore, linear methods should be limited to identifying damping in specific simple structures that operate under normal conditions [36]. These drawbacks have, in the last decade, contributed to the rapid development of nonlinear damping methods [37,38].

Damping identification is a challenging task [39] performed by developing a variety of analytical [40,41,42] and experimental methods of linear and nonlinear systems [43,44,45]. Moreover, NDI is a practical aspect being conducted to prevent structural failure and tragic events caused by structural damages [46,47]. In addition to enhancing the safety and maintenance of key structures, it also contributes to the control of systems and predicting structural systems responses under nonlinear damping properly [48,49].

NDI for dynamic systems can be classified into seven categories: Linearization methods, time-domain methods, frequency-domain methods, time-frequency methods, modal methods, black-box modeling, and model updating methods [1]. Classification can also be made from other perspectives, for example, parametric and non-parametric [50,51]. An example of linearization methods is the equivalent linearization approximation (ELA), which is a common method used in applications such as a spring-suspended sectional model system. ELA is utilized for bridges and aeroelastic systems and dampers and shock absorbers used in control systems [52].

The time-domain methods are well-known methods such as the log decrement method used in lightly damped systems and aeroelastic systems [53,54]. In addition, the Hilbert transform (HT) is used to analyze vibration systems [55], heat exchangers, and damage detection of reinforced concrete (RC) structures and composite materials [56,57]. The frequency-domain methods are known by their mathematical simplicity and ability to provide insightful interpretation [58]. For example, harmonic balance nonlinearity identification (HBNID) was employed in systems such as civil engineering, actuators, bioengineering devices, sensors, and robotics [59]. Furthermore, the frequency response functions (FRFs) have been widely utilized to study nonlinear damping in adhesive joints [60,61].

Time-frequency methods, e.g., those based on continuous wavelet transform (CWT), are powerful tools used in applications such as vibration absorbers that are broadly used in naval architecture, rotor-bearing systems, and constructions [62]. In addition, HT was employed in applications such as unbalance of rotating machines, ship movement control, and damage detection of RC beams based on free vibration measurements for nonlinear damping determination [63]. Modal methods are considered particularly useful in the field of structural dynamics and damage identification. The resonant decay method (RDM) was applied to investigate the nonlinear damping in civil, aircraft, and various types of dampers [23]. The wavelet transform (WT) was used in instantaneous damping coefficient identification for damage detection in concrete, automotive, aerospace, and simple built-up structures comprising two bolted beams [64]. Black-box modeling (BBM) is an accurate and efficient method in describing the dynamic behaviors of structures. For example, a fuzzy wavelet neural network (FWNN) was used for nonlinear identification in systems such as vehicle magnetorheological (MR) fluid dampers, aeroelastic systems, modern industries, control systems, and military and defense equipment [65].

**Figure 1:**
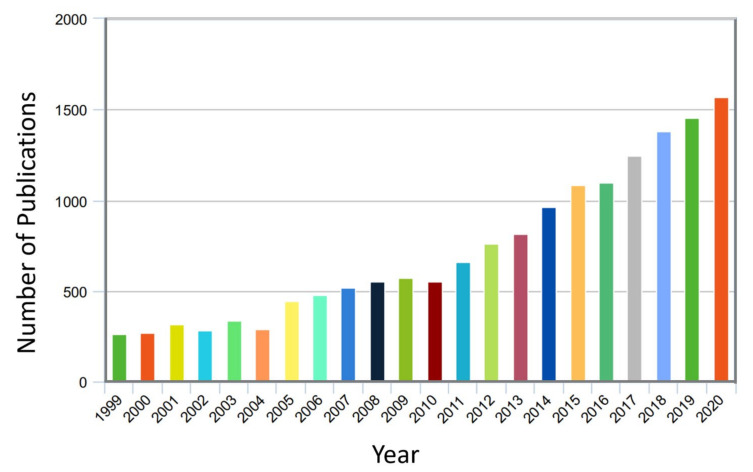
The number of publications for nonlinear damping studies (According to the Scopus engine system in the duration from January 1999 to November 2020).

Model updating methods include, for instance, the identification of structural damping using the FRF-based model updating method and damping identification for accurate prediction of the measured FRFs using finite element updated models of the mechanical system [66,67]. Recently, several studies were conducted on dynamic systems. These studies showed the high feasibility of the NDI methods compared to the linear methods because they give more reliable results despite their difficulty [1]. The nonlinear research focuses primarily on the development of efficient and functional methods for reacting to nonlinear structural damping as a fundamental scientific and technological problem. A survey of available research in the engineering community has provided many studies of nonlinear damping, as shown in Figure 1. It is expected that interest from researchers in this field will continue.

To summarize, NDI methods are essential and emerging methods leverage on their importance in structural dynamics in general. Such methods are able to provide crucial aspects and useful tools to track structural changes and dynamic alterations. Moreover, this can deliver potential future research in the field of structural dynamics as well as damage identification.

This review paper discusses the progress of NDI methods based on available literature in terms of surveying possible methods and applications. Section 2 offers a critical review of the recent nonlinear damping methods based on previous studies to identify the role and placement of NDI methods. Nonlinear damping methods are also discussed using their theoretical principles and schematic diagram. The second section is followed by an overview of possible applications of nonlinear damping methods in many different fields. At the end of this work, a summary and concluding remarks are presented.

## 2. Nonlinear Damping Identification Methods

**Figure 2:**
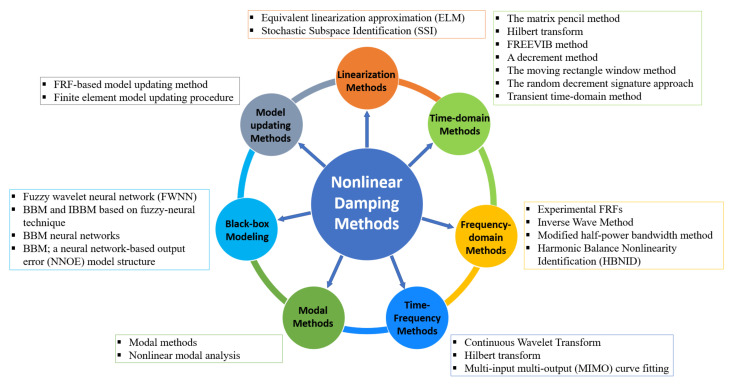
Nonlinear damping identification methods.

The early study on the identification of nonlinearity of structural models can be traced back to 1970s [1]. The objective of this section is to survey the NDI methods that evolved over the last few years as a result of the development of novel industrial materials and structures [68]. Various nonlinear factors have led to different systems’ behaviors, which means that each system has unique behavior and therefore requires a different approach [69]. Relevant studies were first focused on single-degree of freedom (SDOF) systems [70]. Then, with the advancement of computational techniques, studies were extended to more complex, multi-degree of freedom (MDOF) systems [71,72,73]. Specific structural types under investigation include bridges, tall buildings, aircraft, etc. [74]. Apart from previous review articles that are mainly about nonlinear system identification [1,2,23], the focus of this study resides on the development of various NDI methods. The methods can be classified into seven categories, as shown in Figure 2.

### 2.1. Linearization Methods

The linearization method is a process whose objective is to approximate a nonlinear system that is described by a nonlinear differential equation with a linear one for ease of processing. Equivalent linearization (EL) is a widely used approximation method for dynamic system analysis [75]. Krylov and Bogoliuboff introduced the first linearization process of deterministic systems. The stochastic systems approach was then extended by Caughey. Then, some expanded versions of the equivalent linearization method have been established. The Equivalent Linearization method is considered the most accessible tool extensively used for analyzing nonlinear stochastic problems, but its accuracy depends on the averaging process [76]. Other classical methods include the Fokker-Planck equation, moment closure, stochastic averaging, perturbation, etc. However, they are limited to relatively straightforward and specific nonlinear systems, and some of them are computationally expensive, mainly when applied in MDOF systems [77].

**Figure 3:**
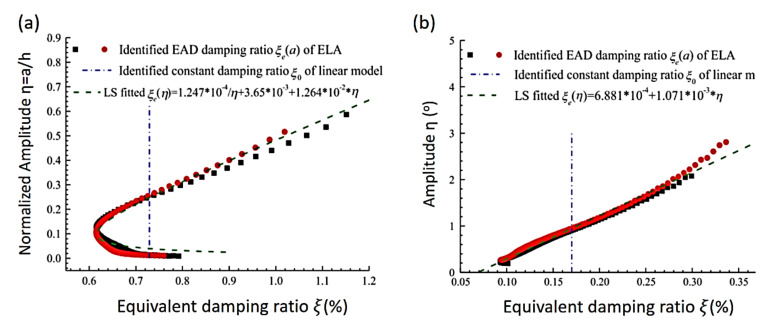
Comparison of identified equivalent amplitude-dependent damping and natural frequency of the spring-suspended sectional models. (**a**) Bending mode; (**b**) torsional mode [81].

Wang and Low [78] proposed a reliable EL method to predict the response of nonlinear systems with viscous damping subject to impact. Moreover, the influences of nonlinearity and viscous damping on the safety of packaged products were discussed. Additionally, coefficients such as the damping ratio in the cushion system were investigated. According to their analysis, both the nonlinear properties and viscous damping afforded are proven to be positive factors able to minimize the rigid impact to some extent. Bajrić and Høgsberg [79] presented an approach for output-only system identification. This method is effective for analyzing the random responses with SDOF oscillators under hysteretic damping. The Bouc-Wen model was used to take advantage of the restoring force to derive the model of an equivalent linear relaxation. The identification was carried out in the state space, where the derived linear relaxation damping model replaced the hysteretic system model. The equivalent linear model proposed that the response motion is harmonic with a slow variation of phase and amplitude, and thus the method was restricted to tackle narrow band response. To study a spring-suspended sectional model system of bridges, Gao et al. [80] developed an EL approach to improving the precision of measuring self-excited force in the sectional model test. The effect of added damping and stiffness on the free decay response at zero wind speed state was explored. Based on their findings, the nonlinear characteristics associated with the influence of the added damping influences are much more significant than that associated with the influence of the added stiffness. In another work, Gao and Zhu [81] proposed an approach where the spring-suspended sectional models (SSSM) were used to evaluate the equivalent amplitude-dependent damping ratio and frequency. The equations of the ELA are derived by applying a multiple-scale method to represent the mechanical nonlinearities in the first-order approximate sense. The proposed ELA and nonlinear system identification methods are then found to be accurate enough to model the mechanical nonlinearities of the proposed system. Figure 3 shows a comparison of identified equivalent amplitude-dependent damping and natural frequency of the SSSM under bending and torsional modes. The nonlinear behavior of damping ratios originates from complicated energy-dissipative mechanisms, such as material damping, Coulomb friction, viscous damping, and nonlinear damping caused by additional SSSM system dampers. In an ELA, weak nonlinear response of a SDOF system is considered as a perturbation on the responses of undamped oscillators, so a nonlinear motion equation that governs the free decay response of the system is presented as follows:

(1) $$\ddot{q}+\varepsilon f\left(\dot{q},\;q\right)+\omega_{0}^{2}q=0$$

where q is the displacement; $\dot{q}$ is the velocity; $\ddot{q}$ is the acceleration of the sectional model; $\omega_{0}$ is the circular frequency; $\varepsilon f\left(\dot{q},\;q\right)$ is the generalized nonlinear force; and ε is a small factor showing that the previous term is a small quantity; $\omega_{0}^{2}q$ is the linear restoring force. Equation (1) can be solved based on the Krylov–Bogoliubov averaging approach. An ELA method was then applied to model the physical nonlinearity of a weak nonlinear system by using a damping coefficient D(A) and a restoring force coefficient S(A),

(2) $$\ddot{q}+D\left(a\right)\dot{q}+K\left(a\right)q=0\;\left(\varepsilon^{2}\right)$$

and by inserting the equivalent viscous damping ratio and frequency, Equation (2) can be represented as:

(3) $$\ddot{q}+2\omega_{e}\xi_{e}\left(a\right)\dot{q}+\omega_{e}^{2}\left(a\right)q=0\;\left(\varepsilon^{2}\right)\;\;\;\;$$

where $\xi_{e}\left(a\right)$ is the equivalent amplitude-dependent damping ratio; $\omega_{e}\left(a\right)$ is the equivalent circular frequency.

Both nonlinear damping and frequency depend on amplitude and are accurate in modeling the physical nonlinearity of a weakly nonlinear spring-suspension system.

**Figure 4:**
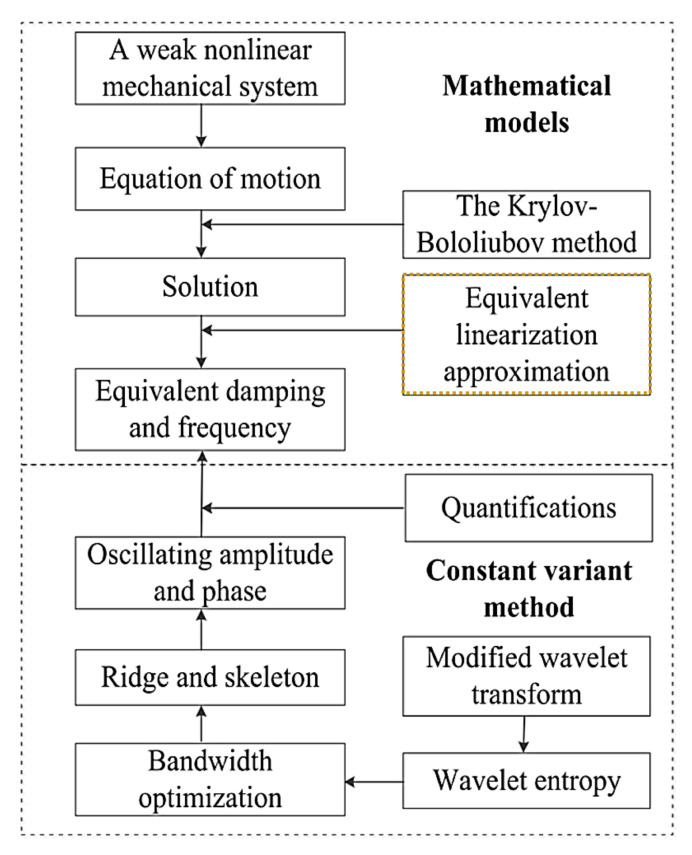
A diagram for nonlinearity identification of weakly nonlinear systems [82].

Recently, Chen and Tse [82] proposed an enhanced method to determine the physical nonlinearity of weakly nonlinear spring suspension systems. The method was effectively implemented in hybrid aeroelastic pressure balance (HAPB) systems. In the HAPB system, the frequency and damping associated with a linear model are constant and cause major differences in predictions of response due to ignorance of the system’s slowly changing characteristics. The solution of a proposed system is obtained by deriving the averaging method of Krylov–Bogoliubov and the ELA method, as shown in Figure 4.

### 2.2. Time-Domain Methods

In time-domain methods, analyzed data are in the form of time-series during the identification process [83]. The benefit is the simplicity in data analysis without much cost of time and effort since the time history of data can always be acquired directly [84].

Typical work is introduced by Jacobson et al. [85], where they evaluated the damping identification methods using time-domain simulation based on an aeroelastic system for the applications of flutter constraints to gradient-based optimization. Several time-domain methods were applied with CFD-based methodology. The matrix pencil method was demonstrated to be the most effective approach in estimating damping over a set of input signals. In another study of heat exchanger tube arrays, Eret and Meskell [86] investigated the validity of two identification methods applied in a SDOF fluid elastic system by using experimental data. The free vibration analysis (FREEVIB) method was used to identify nonlinearity and was then compared with the nonlinear decrement method. According to the results, the nonlinear decrement method produced more accurate results. Nevertheless, its deficiency was that the functional form of the system needs to be predetermined. Meskell [87] presented a technique for simultaneously evaluating different types of nonlinear damping and viscous damping. This technique relies on successive peak decrements in the transient system. The study was restricted to SDOF systems. This technique was optimized based on the consideration that the system was weakly nonlinear, lightly damped subject to linear, and cube damping. The accuracy of this method was demonstrated using simulated responses. It was concluded that the method was promising for a lightly damped system using experimental data, particularly in fluid elastic systems. Frizzarin et al. [88] developed an approach for damage detection in a concrete structure. The approach relies on the analysis of nonlinear damping extracted from structural vibration responses. The feasibility of the approach was demonstrated using a large-scale concrete bridge model suffering from seismic damage caused during shaking table tests. Nonlinear damping was successfully identified by random decrement signature approach based on its ambient vibration responses. The results showed that the magnitude of nonlinear damping increased along with an increasing degree of seismic damage. Strong correlation between increasing nonlinear damping and degrading structural stiffness was also found.

A method of nonlinear damping analysis using ambient vibration data was developed for baseline-free damage detection in RC structures. Viscous and friction damping models were combined to obtain the envelope of free vibration response of the structure, as shown in Equation (4):

(4) $$a\left(t\right)=\;x_{0}\left[\left(1+\frac{\gamma }{\zeta }\right)e^{-\zeta \omega t}-\frac{\gamma }{\zeta }\right]$$

where $x_{0}$ is the initial amplitude; ω is the natural frequency; ζ and γ are the damping ratios for viscous and friction damping, respectively.

**Figure 5:**
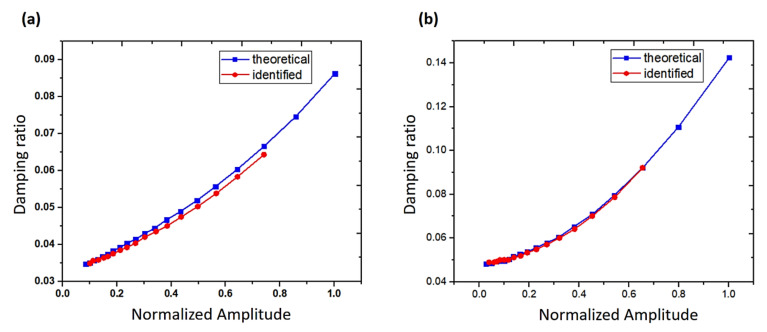
Curves of damping ratio versus amplitude. (**a**) Exponent relationship; (**b**) quadratic relationship [89].

**Figure 6:**
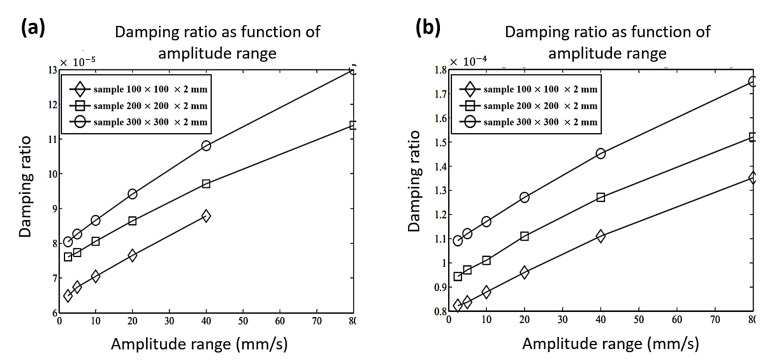
Damping ratio as a function of (**a**) specimen response amplitude (first mode shape); (**b**) specimen response amplitude (second mode shape) for three specimen sizes [90].

Another time-domain method was proposed by Wu et al. [89] to enable simultaneous identification of nonlinear damping and Coulomb friction in mechanical systems. A moving rectangular window method was introduced based on nonlinear damping properties. Different models of nonlinear damping in a SDOF system were studied, with a constant amount of Coulomb friction. The simulation results showed that the proposed identification technique was efficient and applicable. Furthermore, the identification precision of nonlinear damping was higher than that of the force of Coulomb friction. Figure 5 presents the nonlinear relationship between the damping ratios and amplitudes in terms of exponential and quadratic functions, respectively. The accuracy of determining the quadratic damping is the highest compared to that of determining other types of nonlinear viscous damping. In the quantification of unwanted effects on the overall measured damping of steel alloys, Vanwalleghem et al. [90] identified external damping sources in damped material by applying transient time-domain methods and introducing an effective damping test setup configuration. The results showed that the value of damping was dependent on both the specimen size and level of excitation. In addition, damping will increase with enlarged response amplitudes regardless of the sample sizes, as illustrated in Figure 6. Moreover, the damping capacity differed from one steel alloy to another. Therefore, one type cannot be generalized to others. In another related work, Baştürk et al. [91] studied the nonlinear dynamic response of hybrid laminated composite plates under the influence of blast load with damping involved. The effects of factors such as damping ratio, aspect ratio, and various values of peak pressures were studied. The thermal effect of the blast wave was avoided throughout the analysis. The results showed that the amplitude of the damping ratio played a significant role in the deflection of the plate and the frequencies. The vibration amplitude decreased in a short time due to the damping effect. Recently, Feldman and Braun [92] presented promising experimental methods for the identification of nonlinear damping and stiffness in a vibration system. The methods were based on measuring inputs and outputs in the time-domain and the implementation of the Hilbert transform of the measured signals under free and forced vibration states. Based on their findings, the approach of nonlinear characteristics representation was accurate and efficient.

### 2.3. Frequency-Domain Methods

The frequency domain-method is featured by handling data in the form of spectra or FRFs throughout the identification process [93]. Some frequency-domain methods have been reviewed in technical literature in the past years [94,95]. These methods show advantages such as ease of computation and the ability to give some explanations of nonlinear systems [96]. Unlike time-series signals, the data processed in the frequency domain could take various forms, such as Fourier spectra, power spectra, or any other form [97,98]. Since data analysis in the frequency domain only focuses on a specific frequency range, the computational burden can be reduced, and a large number of nonlinear parameters can be calculated precisely and effectively [23].

**Figure 7:**
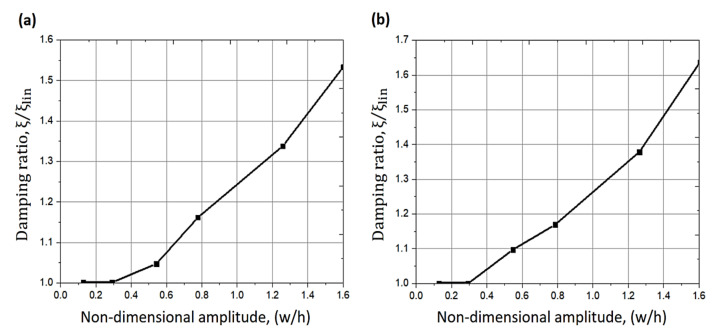
Changes of damping ratio with respect to the amplitude of vibration (**a**) using model 1, (**b**) using model 2 [101].

Sun et al. [99] presented a modified method for damping identification for a nonlinear stiffness structure based on the well-known half-power bandwidth method. The formula was verified by using numerical simulation. The procedure has been validated by applying a hard coating specimen with soft nonlinearity, and the damping parameters of the structure were acquired under various exciting levels. Thothadri et al. [100] extended a nonlinear system identification method named harmonic balance nonlinearity identification (HBNID) to the MDOF fluid-structure systems. Two theoretical models were examined using this extended method. The results showed that HBNID worked well in determining unknown parameters if the model structure was identified. In another related study, Balasubramanian et al. [101] conducted experimental and numerical investigations to determine the increase in damping with the amplitude of the vibration of a rubber plate using three different dissipation models. The nonlinear responses were measured utilizing a laser Doppler vibrometer. According to the results, the increase of damping was around 60% when the vibration amplitude is 1.6 times the plate thickness. The dissipation determined from various models was examined as it confirms the predominant nonlinear nature of damping as a function of the amplitude of vibration, as shown in Figure 7. To reduce the vibration in vehicles for the comfort of occupants, Ho et al. [102] investigated, experimentally and numerically, the vibration isolation using the nonlinear damping performed by a MR damper. The frequency-domain technique was adopted, as the efficiency of the isolation system could be assessed over a wide range of frequencies. Experimental and numerical results showed that a good effect of vibration isolation around resonance regions and high frequencies was achieved, and hence significantly improved conventional dampers’ performance.

The equation of motion representing the nonlinear viscous damping of a SDOF vibration isolation system can be expressed as:

(5) $$M\ddot{y}\left(t\right)+Ky\left(t\right)+F_{c}=-M\ddot{u}\left(t\right)$$

where $y\left(t\right)=x\left(t\right)-u\left(t\right)\;$; M is the mass; K is the spring stiffness; $F_{c}$ is the nonlinear damping force given by

(6) $$F_{c}=\mathrm{sign}\left[\dot{y}\left(t\right)\right]C_{n}{\left|\dot{y}\left(t\right)\right|}^{n}$$

where $C_{n}$ is the constant of the nonlinear damping; 0<n≤1, is the damping exponent; $u\left(t\right)$ is a sinusoidal input displacement specified by $u\left(t\right)=A\;\sin\left(2\pi ft\right)$ with frequency f and amplitude A.

**Figure 8:**
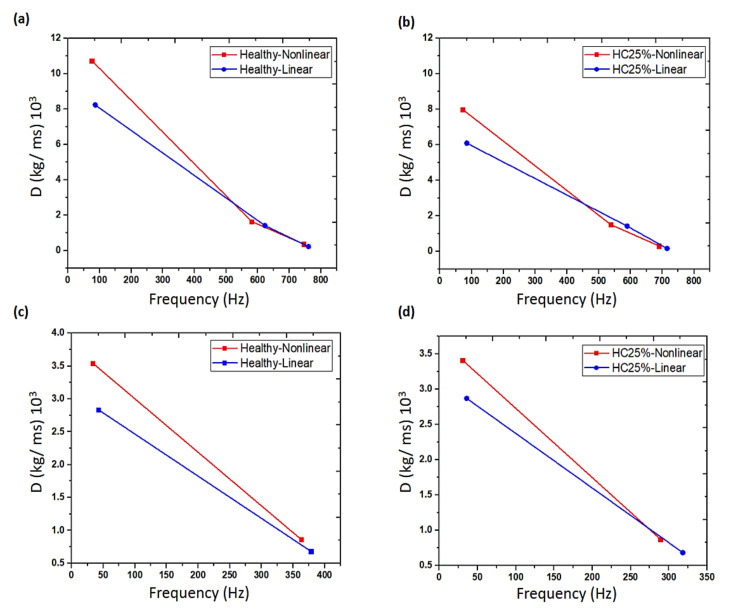
Identified effective damping coefficients for linear and nonlinear areas, for the intact and damaged bonds (**a**,**b**) with bending modes, (**c**,**d**) with shear modes [103].

**Figure 9:**
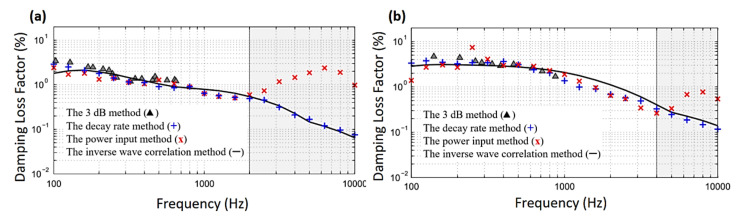
Effect of frequency on experimental damping loss factor of (**a**) a thin composite panel, (**b**) a thick composite panel [104].

Pazand and Nobari [103] investigated the effects of damage on the effective damping of viscoelastic adhesives using the inverse-eigen sensitivity identification method for adhesive behavior in the linear and nonlinear regions. Results showed that the adhesive damping decreased when frequency decreased. Additionally, debonding damage had an adverse influence on adhesive damping, and the reduction (softening) became more important as the rate of damage increased. Figure 8 shows that the effective adhesive damping decreases with increasing frequency in both linear and nonlinear regions. In addition, the reduction pattern is different for various modes such as bending and shearing modes. Cherif et al. [104] presented a damping loss factor assessment approach for two-dimensional structures based on the measurement of the displacement field by using a laser vibrometer. This approach was then compared with the other three approaches: i.e., half-power bandwidth method, decay rate method, and steady-state power input method. From the results, the proposed inverse wave approach was accurate and reliable for the evaluation of the wavenumber and damping loss factor. Figure 9 shows that at mid frequencies, the three methods agree well, but at low frequencies about (100–300) Hz, the power input method shows some discrepancy compared to other methods. The explanation is that these frequencies have a low number of modes. At high frequencies, the damping loss factor evaluated by both the decay rate and the inverse wave methods was in good agreement. Roncen et al. [105] experimentally and numerically analyzed the nonlinear rubber isolator, which was subjected to two random excitations, i.e., the harmonic and broadband excitations. The relation between the stiffness and the damping versus the amplitude of the relative displacement of the rubber isolator was investigated. Nonlinear vibration prediction of the beam exposed to random excitation was conducted by adopting the harmonic balance method and shooting method. According to the comparison between the experimental and numerical investigations, it was observed that there were functional correlations between harmonic and broadband random excitations. This demonstrates the validity and efficacy of the rubber isolator modeling as well as the proposed nonlinear methodology. Recently, Colin et al. [106] investigated many nonlinear quadratic damping features of cantilever beams under harmonic base excitations. The frequency-domain identification techniques were used to identify the linear and nonlinear modal damping coefficients.

### 2.4. Time-Frequency Methods

Recently, applications of time-frequency domain methods have become more frequent and widespread than time-domain and frequency-domain methods [107]. These methods detect damping through common temporal and frequency characteristics of the responses of the vibrating structures resulting from the analysis using time-frequency methods [108,109]. Several methods have recently been presented to perform time-frequency analysis [110,111,112]. One of the most robust approaches is the continuous wavelet transform, which has been progressively utilized for NDI in different applications. A common property of nonlinear vibrations is that, according to the form of nonlinearity, both instantaneous natural frequency and damping coefficient may become time functions [113].

**Figure 10:**
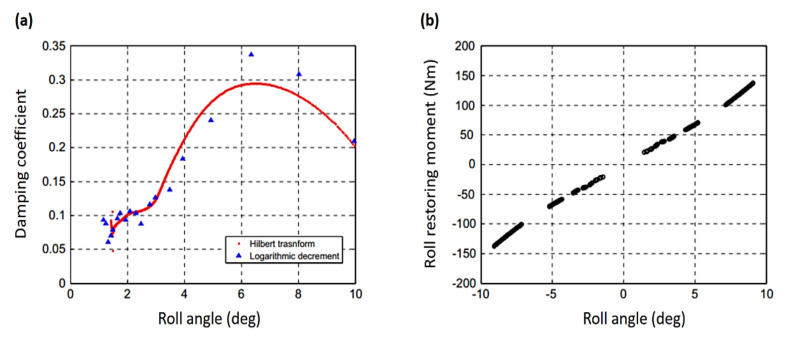
The floating production storage and offloading identified dynamic parameters with respect to the roll angle. (**a**) Damping coefficient; (**b**) restoring moment [114].

In the field of ships and floating bodies, Kim and Park [114] predicted the nonlinear damping and restoring coefficient of a floating production system via the Hilbert transform of free decay signal obtained by a free-roll decay experiment. A comparison was made between the present method and the traditional logarithmic decrement method in the performance of damping coefficient identification. The damping and restoring moment’s nonlinear coefficients were successfully obtained. Figure 10 shows some fluctuations at a small roll angle; however, damping can only be approximated using a quadratic model within a roll angle range of around 6 rad; beyond this range, the damping coefficient decreases. This is owing to the vortex memory effect nearby the bilge keel. The presented technique shows greater accuracy than the logarithmic decrement, especially when the nonlinear terms are combined in the restoring term.

**Figure 11:**
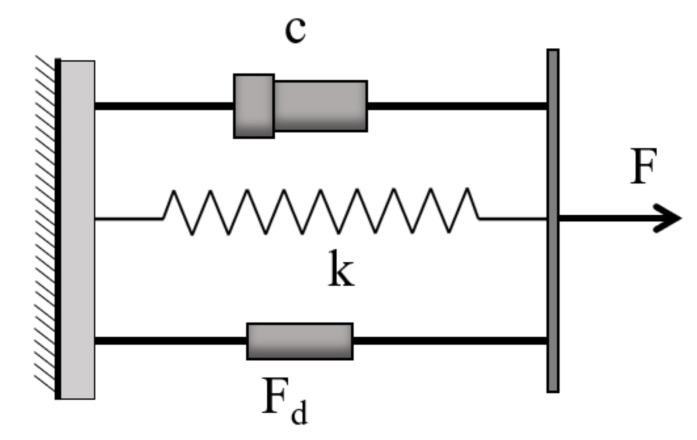
The combined damping model [115].

Franchetti and Modena [115] developed a damage detection technique for precast Prestressed reinforced concrete (PRC) structural members based on free vibration experiments and NDI. Three precast specimens of the PRC beam were tested. The dynamic responses were analyzed by using various methods, including the multi-input multi-output (MIMO) curve fitting and the HT method. The actual energy dissipation mechanism of the PRC beams was represented by a proposed nonlinear quadratic damping factor associated with actual damage levels. The results revealed that the quadratic damping factor could be used efficiently to detect damage due to its high sensitivity. However, the effectiveness of this approach might be constrained by the difficulty of obtaining a free structural vibration response. The pure viscous damping and polynomial damping are combined in the proposed model, as displayed in Figure 11. The most popular form of the force of polynomial damping is quadratic:

(7) $$F_{d}=-d\cdot \dot{x}\cdot \left|\dot{x}\right|$$

where d is a constant, and the value of the absolute velocity is added to ensure that the force is at all times velocity opposed.

The equation of motion of the combined viscous and quadratic system can then be represented as follows:

(8) $$m\ddot{x}+c\dot{x}+d\dot{x}\left|\dot{x}\right|+kx=0$$

where *m* is the mass, *c* is the viscous damping coefficient, and *k* is the stiffness,

(9) $$a\left(t\right)=\frac{\left(a_{0}c_{1}\right)\;\cdot \;e^{-c_{1}\;\cdot t}}{c_{1}+a_{0}c_{2}\;\left(1-e^{-c_{1}\;\cdot t}\right)}$$

where *a* is the amplitude of oscillation, *a*_0_ is the initial amplitude, *c_1_* and *c_2_* are constants, and *t* is the time. The exact solution of pure viscous damping ($c_{2}=0\;$) is formulated as follows:

(10) $$a\left(t\right)=a_{0}\cdot \;e^{-c_{1}\;\cdot t}=a_{0}\cdot \;e^{-\xi \omega t}$$

Tang et al. [116] experimentally investigated the assessment of the nonlinear vibration absorber parameters from free vibration tests. HT was utilized to estimate both instantaneous amplitude and damped natural frequency. Then the stiffness and damping were determined for a nonlinear vibration absorber. By comparing the present method and Restoring Force Surface (RFS) method, it was shown that there was a difference of only about 13%. Chandra and Sekhar [117] presented a nonlinear damping estimation approach in a rotor-bearing system using the CWT based method. Two different nonlinear damping models were examined. The free vibration signal envelope was obtained by employing the wavelet-based approach. The validity and applicability of the proposed method were reported using the acquired signals from the experimental results. Joseph and Minh-Nghi [118] used wavelet transform in the identification and quantification of damping in a nonlinear oscillator based on free decay response. Two methods based on wavelet transform have been used; firstly, the cross-section of the wavelet transform, secondly, the ridge and skeleton of the wavelet transform. The proposed method herein was used to study a nonlinear SDOF oscillator exclusively. Numerical results showed that the method is accurate in the estimation of natural frequency and damping coefficient, even with noisy data.

Curadelli et al. [119] introduced a new scheme for structural damage detection using the instantaneous damping coefficient identification via wavelet transform. Based on their findings, the damping of various structures is significantly affected by the existence of damage. Evidence for damage effects on the dynamic characteristics of the RC frame has been provided. Therefore, the structural damping parameters can be used as damage indicators due to their high sensitivity to damage existence. It has also been shown that the wavelet transform can be used to determine the damping through a structural response to free vibration.

In another related work, Heller et al. [120] experimentally analyzed the influence of mechanical joints and their functional parameters on the dynamic behavior of built-up structures. The equivalent modal parameters based on the application of wavelet transformation used to the free-decay response were adopted to describe the expected nonlinear dynamic behavior. Experimental results demonstrated that the frictional joints are the major sources of energy dissipation during the relative motion of substructures in the built-up structure. Furthermore, the negative damping capacity can be significantly increased by widening the interface area.

**Figure 12:**
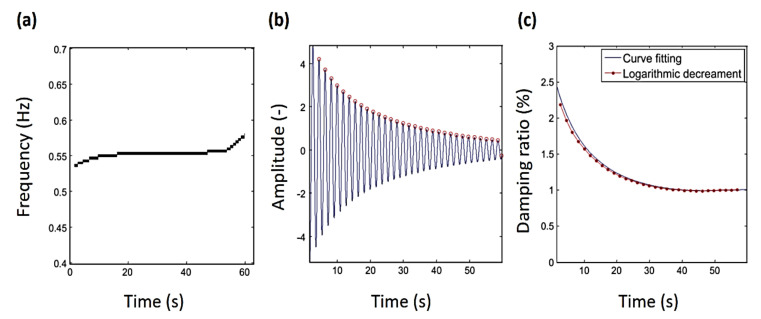
The first mode identification results of vibration of the analyzed tuned liquid column damper (TLCD): (**a**) Natural frequency, (**b**) vibration response, (**c**) damping ratio [121].

Recently, Dziedziech et al. [121] examined the dynamics of a tuned liquid column damper (TLCD), where the time-varying characteristics of nonlinear damping and other properties were identified. CWT was used for mode separation, and then the recognition of the instantaneous damping ratio of the first mode of vibration was conducted. The findings showed that the damping ratio was nonlinear, time-varying, and based on the level of vibration. The introduced model can be employed to represent the dissipative behavior of the first mode of vibration of the tuned liquid column damper. The time-varying damping was successfully recognized utilizing the combined envelope analysis, curve fitting, and logarithmic decrement, as shown in Figure 12.

### 2.5. Modal Methods

Modal characteristics are considered highly important in the process of designing linear engineering structures [122]. Typical structural modal parameters are natural frequencies, mode shapes, and damping [123]. It is a beneficial tool used in the study of the dynamic behavior of various structures around their resonance state. This linear approach is considered to be mature and widely used [124]. On the contrary, methods for identifying nonlinear modal are in one of the promising directions of recent research in the field of structural dynamics [125,126]. Some related approaches have been introduced in the surveys in 2006 and 2016 [1,23]. Noteworthy progress has been made over the past two decades, so nonlinear modal identification is a very active field of study [23].

**Figure 13:**
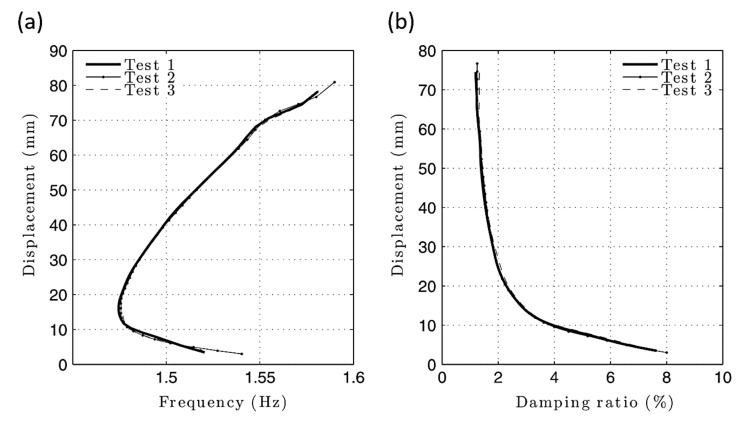
The estimated skeleton curves from the experiment: (**a**) Frequency (Hz); (**b**) damping ratio (%) [129].

In order to improve engineering designs, reliability, and performance of structures, Mezghani et al. [127] investigated the dynamic characteristics of an isolator consisting of stiffness and damping elements. The investigations were performed under various excitations to promote the minimization of the vibration transmissions using modal methods. The proposed nonlinear identification method allows designers to predict nonlinear dynamic behavior via experimental data. Results revealed that the present technique is valid and applicable in determining the nonlinear parameters of a metal mesh isolator. The nonlinear damping may also be suppressed as the base excitation amplitude increases. Naylor et al. [128] introduced the resonant decay method to estimate the MDOF nonproportional damped systems. Though there were some imperfections, the method performed well and produced an acceptable modal damping matrix. The results indicate that there were small errors in the estimation process. Londoño et al. [129] introduced a method to extract the backbone curves of the lightly damped nonlinear systems utilizing a modified resonance decay method. The experimental and numerical results show that the proposed procedure can offer an accurate estimation of damping ratios. Moreover, results indicate that the small damping ratio lower than 5% does not influence the estimated instantaneous frequency of the nonlinear system, as shown in Figure 13.

By analyzing the envelope of decaying response, the dissipation properties of the system can be evaluated. For a classic, well-known SDOF system with a common equation

(11) $$\ddot{x}+2\xi \omega_{n}\dot{x}+\omega_{n}^{2}x=0$$

where $\ddot{x}$ is the acceleration; $\dot{x}$ is the velocity, x is the displacement, ξ is the damping ratio, $\omega_{n}$ is the natural frequency.

In terms of system parameters and initial conditions, the envelope of the free vibration response can be represented as

(12) $$A\left(t\right)=A_{0}e^{-\xi \omega_{n}t}$$

It should be noted that the operation of damping is accounted for by the exponential term. This expression can be generalized to enable the change of instantaneous frequency and damping, which would occur in a nonlinear system by modifying Equation (12) to be

(13) $$A\left(t\right)=A_{0}e^{-\xi \left(t\right)\omega_{0}\left(t\right)t}$$

The effective damping then could be determined by

(14) $$\xi \left(t_{i}^{o}\right)=\frac{1}{\omega_{0}\left(t_{i}^{o}\right)t_{i}^{o}}\left(\left(\ln(A_{0}\right)-\ln\left(A\left(t_{i}^{o}\right)\right)\right)$$

where $\omega_{0}\left(t_{i}^{o}\right)=2\pi f\left(t_{i}^{o}\right)$ denotes the instantaneous angular frequency. Therefore, the effective damping ratio ξ can be assessed from the envelope tangent slope of the decaying response represented in a semi-logarithmic scale with respect to time.

Krack et al. [130] developed a novel reduced order model (ROM) method for numerical calculation of nonlinear modes of mechanical systems. The study included some types of nonlinearity, such as dissipative and strong and non-smooth nonlinearities. The nonlinear modal characteristics were utilized to evaluate the forced and self-excited vibration. The obtained results were in very good agreement with results obtained by conventional approaches. In another related work, Krack [131] proposed an identification method of the nonlinear modes of nonconservative systems. Two methods for nonlinear modal analysis were presented: The harmonic balance method and the shooting method. The results show that the proposed method provides accurate predictions for a broad range of working conditions. However, it is limited to the isolated nonlinear modes and low modal damping ratios as it is also restricted to periodic motions. Peter et al. [132] numerically and experimentally proposed a new technique for the nonlinear modal analysis of non-smooth mechanical systems. The numerical technique was found based on two combined methods. They are the harmonic balance method and the shooting method generating a time-frequency representation. In comparison, the experimental technique relies on a nonlinear phase resonance method. It was found that the numerical results were in good agreement with results obtained by experiments. Scheel et al. [133] developed a new technique based on an experimental procedure for nonlinear modal testing for modern turbine blades. The technique allows the experimental extraction of modal damping ratios in addition to both natural frequencies and deflection shapes as a function of the vibration level. It is simple and more time-effective as its validity and accuracy have been proved through comparison to other methods. It also has high resistance against measurement noise and does not require much prior knowledge of the tested systems or special equipment. In another related work, Scheel et al. [134] designed a new test rig named rubbing beam resonator for experimental nonlinear modal analysis. The proposed method performs the analysis based on the concept of the extended periodic motion in order to obtain the modal characteristics. The results show that the modal damping ratio increases by about 15%, while the frequency decreases by 36% for the first mode of vibration. Recently, Karaağaçlı and Özgüven [135] proposed a nonlinear experimental modal analysis procedure called response-controlled stepped-sine testing (RCT) to extract the nonlinear modal parameters. Many nonlinearities at different places were investigated numerically and experimentally. The numerical and experimental results showed that the proposed identification procedure was efficient and applicable to many applications.

### 2.6. Black-Box Modeling

Nonlinear black-box is a typical model used to characterize nonlinear dynamic behaviors of systems only based on data. Nonlinear black-box modeling is a mapping from past observed data to a regressor space pursued by a nonlinear function expansion type, mapping to the space of the outputs of the system [136]. Nonlinear mapping can be performed by several methods, e.g., wavelet networks, artificial neural networks, and neuro-fuzzy models [137].

Witters and Swevers [138] discussed the black box identification of an electro-hydraulic semi-active damper for a vehicle suspension. For the representation of the complex nonlinear damper dynamics, a neural network-based output error structure has been chosen. It has been shown that a model described using single optimal testing can adequately describe the nonlinear dynamic behavior of the damper throughout its entire working period. Truong and Ahn [139] introduced a nonlinear black-box model and an inverse black-box model to study the magneto-rheological fluid damper. A fuzzy mapping system is used for the identification of the damper properties. A neural network method was used to improve the accuracy of the model by decreasing the modeling error function. A series of investigations were performed to verify the effectiveness of the models on two systems using the same damper. Khalid et al. [140] investigated a small-scale MR damper model with the valve mode mechanism using a dynamic recurrent neural network modeling method to generate its hysteretic nonlinear response. The modified model of Bouc-Wen was used to generate the training data to construct the numerical model. The results show that the proposed model is effectively capable of accurately predicting the MR damper response over a wide range of operating conditions. Recently, Dou et al. [141] investigated a nonlinear identification method based on a fuzzy wavelet neural network for a nonlinear aeroelastic system. Nonlinear damping was considered in the aeroelastic system identification, and a new FWNN structure was introduced. Numerical simulation shows that the fuzzy wavelet neural network attained a high level of accuracy and effectiveness.

### 2.7. Model Updating Methods

Finite element (FE) model updating is an operation intended to calibrate the FE model of a structure for matching the experimental and numerical results [142,143]. This process aimed to obtain an accurate model that is able to reproduce the measured data [144,145,146]. Despite the rapid development of computer-aided methods, numerical models still need to be compared with empirical data to update them in order to improve their accuracy and reliability [147].

Arora et al. [148] proposed a new scheme for damped FE model updating to improve the FRF compatibility based on the implementation of the damping identification method. Two steps are carried out, the first is the updating of mass and stiffness matrices using the response function method, and the second is the identification of the damping matrix following the first step. The results show that the updated model is able to predict the measured FRFs.

First, the eigenvalue problem related to a viscously damped system written as

(15) $$\left[M\;\lambda_{i}^{2}+C\lambda_{i}+K\right]\Phi_{i}=0$$

where *M*, *C*, and *K* are the mass, damping, and stiffness matrices, respectively; $\lambda_{i}$ and Φ are the complex eigenvectors.

Furthermore, the damping matrix can be assessed using the following relation

(16) $$C=-M\left(\Phi \Lambda^{2}\Phi^{T}+\bar{\Phi \Lambda^{2}\Phi^{T}}\right)M$$

where Λ is the diagonal matrix of complex eigenvalues, and the overall bar refers to complex conjugations. The complex eigenvectors are standardized as

(17) $$\Phi_{i}^{T}\left(M\lambda_{i}^{2}-K\right)\Phi_{i}=\lambda_{i}$$

The updated mass and stiffness obtained in the preceding part are exploited to normalize complex eigenvectors.

Recently, Arora [149] introduced a new technique for the identification of structural damping. It is a direct and explicit identification method. The updated matrices of mass and stiffness were used for the identification of the structural damping matrix. Some instances were provided, illustrating the performance of the proposed technique. According to the results, the current technique was accurately proficient in predicting the experimental FRFs of the system with all damping levels. Table 1 summarizes the NDI methods in several application areas, with some strengths and weaknesses of these methods based on previous studies. It also provides a useful means for researchers to compare the different studies and facilitate understanding of the NDI methods.

## 3. Trending Applications

**Figure 14:**
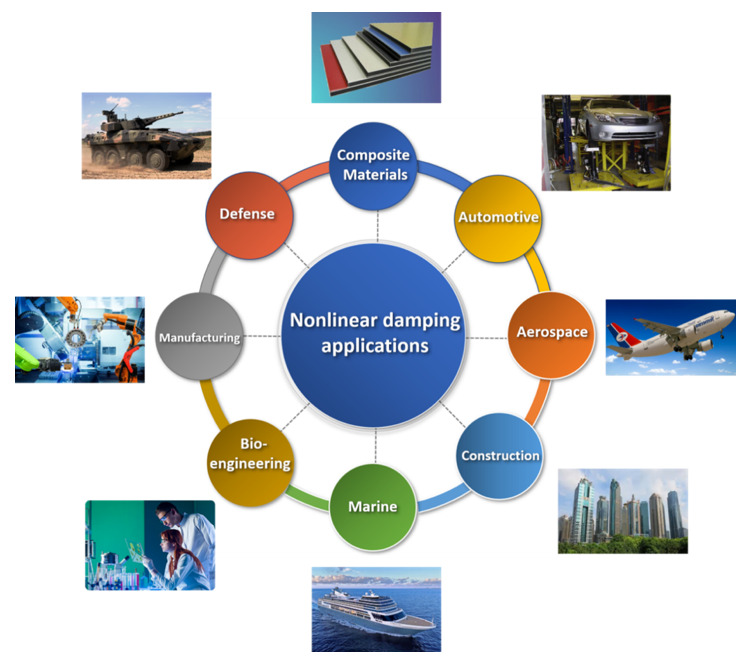
Nonlinear damping applications.

In recent years, many engineering applications have used nonlinear damping to study the nonlinearity phenomenon. This phenomenon has been exploited in the development of various areas of life, such as automotive, bridges, buildings, aerodynamics, marine, defense, and bio-engineering, as shown in Figure 14. This progress would not have been possible without the development of technologies that helped to build and test these applications in terms of safety and reliability [150]. Many improvements have been made to applications such as the use of composites materials and alloys that have good properties that combine safety and performance [151]. Most engineering applications are subjected to unwanted vibrations that should be controlled. Exposure to time-variant loads, such as vehicles, wind, earthquakes, and sea waves, in addition to an imbalance in rotating machines, lead to damage and failure of applications.

As mentioned earlier, there is continuous development in various practical life applications. One of the most important facts that many new materials and applications exhibit nonlinear behavior. Such structures require nonlinear study. This study can accurately predict the behavior of dynamic systems.

Nonlinear damping is one of the most critical and complex aspects of nonlinearity. Researchers are spending more efforts to develop many solutions, theories, mathematical models, and conduct experiments. These efforts were made to accurately predict the dynamic behavior, control the level of vibrations, and avoid problems resulting from excessive vibrations.

### 3.1. Automotive Applications

Several real-world applications of nonlinear dynamic behavior have been reported in the literature. Automotive dampers are one example of complex systems in which their responses are unstable. For example, vehicles are facing changing conditions, which can be specifically expressed on the basis of the frequency. Moreover, automotive dampers are robustly reliant on temperature due to viscosity effects, and hence the variation of the damper response. In automotives, brake squeal, which results from the friction difference between the pads and the rotor, is annoying and considered as an example of an unwanted effect of nonlinearity. The rapid development in the design of automotive technology led to more stability in the performance, comfort, and safety of passengers [152].

**Figure 15:**
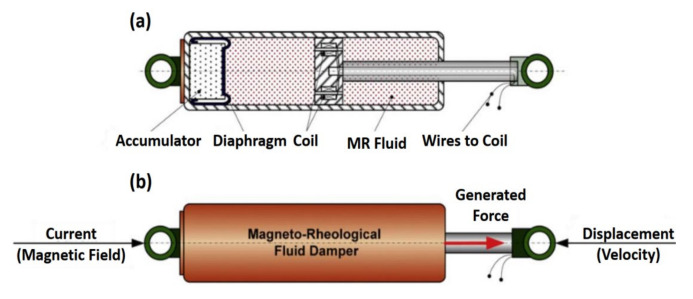
The magnetorheological (MR) fluid damper: (**a**) Hardware structure, (**b**) working principle [139].

Worden et al. [153] considered three nonlinear system identification methods of the suspension system of automotive dampers. The methods are the restoring force surfaces, the nonlinear identification by the feedback of outputs approach, and the nonlinear optimization using a neural network analogy. This study was performed to provide complete insights into the behavior of the systems. In related work, Metered et al. [154] applied an experimental identification procedure based on the black-box modeling, the feed-forward, and recurrent neural networks. The study was carried out to investigate the dynamic behavior of Magnetorheological fluid dampers and the use of the identified parameters in the control of such damper. In another related work, Truong and Ahn [139] presented a nonlinear black-box model and an inverse black-box model to identify the MR fluid damper and applied them to form a new force-sensorless control approach for any damping system. Figure 15 shows a typical MR fluid damper, the hardware structure, and the working principle, which is widely used in many applications such as automotive, aviation, and control. In another research, Salton et al. [155] developed a nonlinear discrete-time control approach for the fast-tracking of quadrotor-like automobiles. A disturbance observer was performed to assist the system in terms of rejecting disturbances and minimizing the effects of unmodeled dynamics. The nonlinear damping was presented to the system using a composite nonlinear feedback control law. Recently, Bonisoli et al. [156] experimentally investigated the influences of the shock absorber of a vehicle chassis on the identification of modal properties and damping matrices via the Layer Method.

### 3.2. Rotors Applications

It is well known that nonlinearity effects are significant to the stability of rotor dynamics. Foundations and supporting structures have substantial effects on the performance of the rotating machine [157]. Accordingly, an equivalent system of a rotor foundation must be determined to reproduce the vibration behavior of the overall system to represent the actual behavior. Squeeze film dampers are often used in current turbomachinery, particularly aero-engine, to control the vibration magnitude of the rotor. Some necessary information, such as fluid forces, are required to predict the characteristics of such dampers [158].

The high-speed turbomachines used in power generation should be power-saving, economical, and have high performance. Using gas-lubricated bearings may contribute to many advantages like lower power loss, reduced vibration, and higher operating speed. This can be achieved by selecting a suitable damping system [159,160].

The study of rotating structures comprises many different challenges, unlike stationary structures. Nonlinear impacts are apparent once the amplitude levels of vibration are high. The main difficulties associated with defining a linear system of rotor-bearing systems have been discussed in many previous studies, unlike nonlinear systems that still need a broader study.

**Figure 16:**
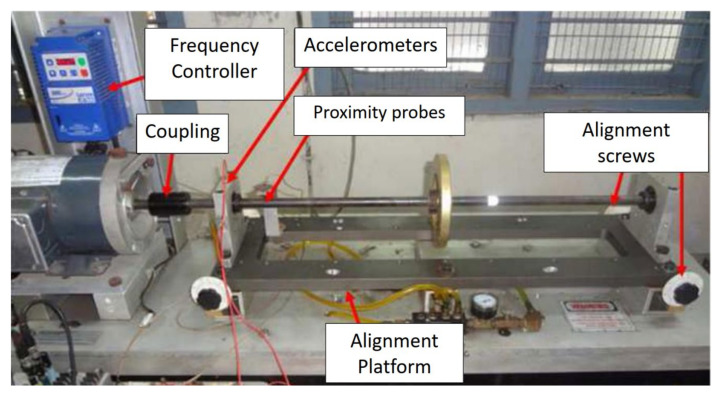
Experimental setup for nonlinear damping identification in rotors [117].

**Figure 17:**
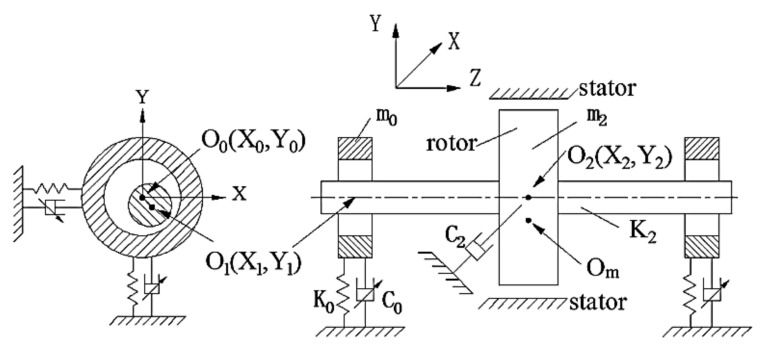
Nonlinear damping suspension in a flexible rotor in journal bearings [165].

Several techniques are available in the literature to determine the effects of nonlinear damping. In recent decades, various theoretical and laboratory experiments to evaluate the effects of nonlinear damping and the behavior of rotor-bearing structures have been carried out. Tasker and Chopra [161] determined the equivalent linear viscous damping properties for a nonlinear damping system from sampled, noise, multi-mode transient response data. The study was carried out through updated versions of the moving-block analysis and sparse time-domain technique. Numerical simulation was used for typical rotor environment representation. They studied two kinds of nonlinear damping, the Coulomb damping and quadratic damping. In a study of turbomachinery, especially aero-engines, Zhang and Roberts [162] developed a novel frequency-domain approach to estimate unknown parameters in nonlinear dynamic systems. The study was conducted through the direct application of the window functions for different terms in the motion equations. The results of simulation tests were carried out on a nonlinear model of the squeeze-film damper showed that the introduced method could provide satisfactory estimations for different unknown parameters. Figure 16 shows an experimental setup for nonlinear damping identification in rotors. In another study, Smith and Wereley [163] analyzed three methods for damping identification for linear and nonlinear helicopter rotor systems from transient experiment data. The methods are the analyses based on a periodic Fourier series decomposition, the FFT-based moving block analyses, and the HT-based method. They evaluated the influences of data block length, error at the assumed frequency, and noise on the precision of the particularized damping parameters. In another study [164], they evaluated three nonlinear damping identification methods for magnetorheological (MR) dampers of helicopter rotor systems using extracted transient data with known levels of Coulomb and quadratic damping. In related work, Yan et al. [165] performed a computational study to investigate the influence of nonlinear damping suspension on the non-periodic motions of a flexible rotor in journal bearings. The effect of nonlinear damping was dependent on the speed of the rotor. The numerical method of a fourth-order Runge-Kutta (R-K) was used in the solution of the dimensionless equations of motions. The proposed method can be used for vibration isolation between the bearing and environment. Figure 17 shows a nonlinear damping suspension in a flexible rotor in journal bearings. Yu et al. [166] introduced a detection method for the identification of the equivalent system of structures with non-proportional hysteretic damping assumptions. The vibration measurement of the structure subjected to harmonic excitation was employed. Yamada et al. [167] numerically and experimentally studied the effect of surface texture on journal bearings’ dynamic properties. The study aimed to investigate the damping and stiffness coefficients of the oil film of the bearings in the rotating system. Recently, Delgado and Ertas [168] introduced the compliantly damped hybrid gas bearings (CHGB) to study the dynamic characteristics performance of a damped gas-lubricated bearing system of turbomachinery applications. The results show that stiffness increases with increasing excitation frequency and rotor speed, whereas damping decreases.

### 3.3. Bridges Applications

Bridges are exposed to many external excitations. Some of these excitations are earthquakes, winds, vehicle loads, chemical, and environmental conditions. These excitations lead to the deterioration of the bridges’ conditions, and consequently, catastrophic collapses due to unrecognized damage during periodic visual inspections [169]. The visual inspection is not enough for damage recognition; therefore, it is necessary to use modern technologies. One of these techniques is the use of damping as a damage indicator due to its high sensitivity to the presence of structural damage. By comparing both intact and damaged cases, we can recognize damages in bridges, where there is a relationship between the damping magnitude and damage levels [170].

During the last decades, several types of research have been carried out to understand the relationship between nonlinear damping and the level of damage to bridges. Frizzarin et al. [88] developed a time-domain damage detection technique for a concrete structure. It utilized nonlinear damping as a damage indicator for RC structural parts. The approach had successfully identified nonlinear bridge damping with seismic damage due to the acoustic vibration reaction through the application of a random decrement signature technique. The nonlinear damping-based approach successfully identified different levels of seismic damage on the bridge model.

In another investigation, Zarafshan et al. [171] determined damping in an operational highway bridge by applying two methods, specifically, the decay of motion direct measurement and the natural excitation method. The research described was intended to demonstrate the efficacy of simplified approaches for the determination of the damping properties of typical highway bridges. In a related study of damage detection of bridges, Dammika et al. [172] proposed an energy-based damping identification method for a steel truss bridge. They analytically and experimentally estimated the damping parameters of the bridge, and therefore the modal damping ratios were analytically estimated.

**Figure 18:**
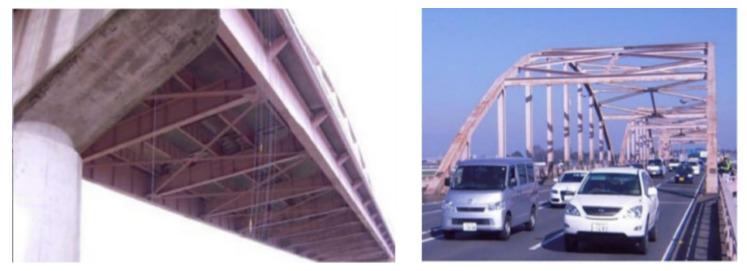
Two views of a steel arch bridge [173].

Recently, Dammika et al. [173] introduced an analytical method based-energy for the evaluation of the modal damping ratios for the bridges and followed by an experimental test on a steel arch bridge. The proposed method has proved its ability for the determination of the damping sources in steel bridges and the contributions to every modal damping ratio and complements the experimental structural health monitoring of bridges. Figure 18 shows a bridge under the influence of moving different vehicles where there is a problem of interaction between the moving vehicles and the structure of the bridge. This movement induces unwanted vibrations that affect the bridge structure over the long-term.

### 3.4. Buildings Applications

**Figure 19:**
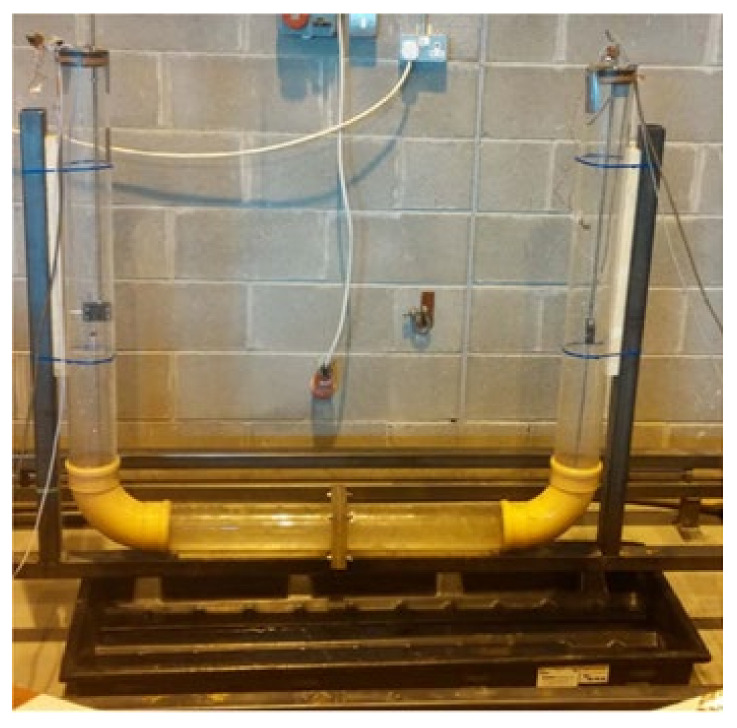
Tuned liquid column damper (TLCD) used in the test of buildings [121].

It is well-known that the dynamic characteristics of many structures, such as high-rise buildings, vary depending on the amplitude of the vibration [174]. Determining damping in structural systems is a very complicated problem due to damping related to many physical phenomena [175,176]. Damping plays an essential role in making tall buildings more flexible and withstanding external influences such as earthquakes and strong winds [177]. Figure 19 shows a tuned liquid column damper (TLCD) used in the damping test of buildings. Such a damper can be used for improving the dynamic of a substructure where it can absorb the energy transferred from the vibrating structure [178]. This type of dampers can be used widely in civil engineering structures and constructions to reduce the effects of earthquakes and wind loadings. Many problems are encountered due to extensive oscillations of tall buildings, such as the discomfort of the building occupants and may lead to severe damage to structures and possibly collapse [179]. Structural nonlinearities occur due to certain causes such as structural damage and joint loosening that disallow the linear analysis of structural dynamics, which means the necessity of inserting the concept of nonlinearity [180].

During the last decades, numerous studies have been made to determine the relationship between nonlinear damping and the level of damage to buildings’ structures. Ling and Haldar [181] proposed a new time-domain identification technique for the evaluation of nondestructive damage of structures. It correctly recognized the stiffness of the structure for both viscous linear and nonlinear damping cases. Furthermore, the proposed method was capable of identifying structures, even with noise-contaminated response data. Noteworthy is a study by Kareem and Gurley [182] that used the random decrement method to estimate the damping in structures focusing on the treatment of uncertainty in its prediction and evaluation. They examined some types of damping sources to structures and the uncertainty treatment in the estimation of damping for real-world applications. In another related work, Huang and Gu [183] proposed an envelope random decrement technique (RDT) for the estimation of nonlinear damping of tall buildings. Three numerical simulations were performed to compare and analyze the performance of the proposed technique in evaluating the amplitude dependence of damping ratio with both conventional RDT and RDT peak. The superiority of the proposed method was proved over the other two methods in assessing the amplitude-based nonlinear damping ratio, as it was also applied to a practical application. In another related study, Béliveau [184] developed a damping identification method in structural dynamics based on modal information within a Bayesian framework. For the calculation of the natural frequencies and damping constants, the modified scheme of Newton-Raphson was used. The actual data of the nine-story steel structure were utilized in the application of the approach.

Recently, Mimura and Mita [185] proposed an automatic evaluation method for obtaining frequencies and damping ratios under the assumption that the information on mass distribution and pattern shapes was available. They tested 40-story steel structural models built with RC. The proposed method was applied to 29-story high-rise steel buildings damaged by an earthquake.

### 3.5. Marine Applications

Marine ships are one of the most important devices of transportation in the current days [186]. There are many marine applications, such as passenger and cargo ships, barges, and warships [187]. These ships are subject to continuous periodic motion as a result of wave flow [188,189]. The motion affects the comfort of passengers and crews, structural safety, and ship controlling and directing [190,191]. The main reason for such behavior is owing to the very high nonlinear characteristics of the sophisticated damping mechanism of the ship, such as the effect of fluid viscosity. Consequently, during the ship design process, nonlinearity must be taken into account. An accurate ship movement forecast allows designers to achieve the requisite dynamic stability.

**Figure 20:**
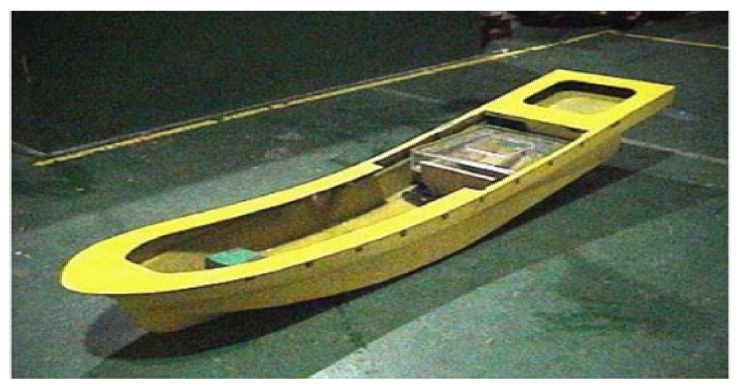
Model of a testing vessel [194].

There has been considerable effort to study this phenomenon; however, understanding nonlinear damping is extremely difficult due to the strong nonlinearity. Golding et al. [192] presented an estimation method for the online identification of nonlinear viscous damping forces for a surface vessel. The approach is based on parameter estimation in conjunction with qualitative data around longitudinally distributed drag parameters extracted from measured data. The proposed method was applied to realistic conditions and provided accurate estimations of the viscous nonlinear drag forces. In other related work, Jang et al. [193] studied the identification of nonlinear roll damping moments of ships containing the same structures. A regularization approach was used to suppress instability. The issue of determining damping was mathematically included in the first type of the integrated Volterra equation between the roll responses and unknown nonlinear roll damping. Figure 20 shows a model of a testing vessel. Jang et al. [194] performed a free-roll experiment for a particular ship to determine the functional form of the nonlinear roll damping. The first kind of the integrated equation of a Volterra-type was mathematically created to identify the nonlinear damping function. The solution instability was suppressed using Tikhonov’s regularization method. Jang [195] identified the nonlinear damping and restoring properties of nonlinear vibration systems in a nonparametric form in which the nonlinear damping is described as a function of velocity only. He introduced the concept of the zero-crossings, which was employed to present a technique for a nonlinear simultaneous identification. In a related study, Han and Kinoshita [196] studied a novel nonparametric and output-only identification method of nonlinear damping. They formulated a stochastic inverse problem for nonlinear damping based on the concept of the stochastic state space. Numerical and experimental investigations were conducted to establish the validity and effectiveness of the proposed method. Recently, Sathyaseelan et al. [197] presented an identification method for nonlinear damping coefficients to a ship roll motion model using the Legendre wavelet spectral method. They made a comparison between the findings obtained using the Legendre wavelet spectral method and the fourth-order R-K algorithm. The proposed approach could be applied to multiple degrees of freedom problems.

## 4. Summary and Recommended Research Directions

Nonlinear damping methods are more accurate and provide a better understanding of the dynamic behavior of real structures and are increasingly used compared to linear methods. In general, nonlinear damping methods are rapidly evolving and increasingly technologically, due to their superior features, with a wide range of uses across all the engineering, vehicles, rotating machines, bridges, buildings, and ships. This critical review discusses the common nonlinear damping methods such as linearization methods, time-domain methods, frequency-domain methods, time-frequency methods, modal methods, black-box modeling, and model updating methods with fundamental difficulties and strengths as well as applications of these methods. This review paper also provides a credible platform for academicians and researchers in this area to understand the basic principles and nuances of these methods. Although there has been much improvement in the development of these methods in recent years to determine nonlinear damping, other aspects still have to be studied to understand this phenomenon. The recommended research directions are summarized to move forward on this topic.

(1) The issue of NDI should be considered in the early design stages as this has an impact on improving the safety and efficiency of engineering structures.
(2) Damping has a higher sensitivity and reliability than natural frequencies and mode shapes to structural damage detection and can be used as a useful indicator for determining damage, which should be further clarified.
(3) Concerning the damping ratio, the instantaneous damping coefficient is a particular property of nonlinear damping, and it is suitable to give an appropriate image that helps in assessing the structural damage caused due to the nonlinearity. Therefore, more attention must be paid to such methods in order to identify the coefficient of the instantaneous damping.
(4) One of the main reasons why using nonlinear damping is more challenging to employ in the process of determining structural damage is the uncertainty in damping evaluation. Therefore, robust and reliable techniques should be developed that can give accurate and reliable results.
(5) Wavelet-based time-frequency techniques for nonlinear damping identification have shown the feature of robustness to noise and usefulness in identifying nonlinear damping. A crucial step towards advancing such techniques lies in overcoming the outstanding matter of choosing optimal wavelets for the analysis.
(6) Damping in composite materials is complicated, as it includes various energy dissipation mechanisms. Besides, composite materials are anisotropic and non-uniform shapes; it needs further study.
(7) In some cases, it is convenient using more than one method to describe the nonlinear damping behavior of structural dynamics accurately. One approach may not give a complete explanation because of many influencing factors on systems. So, it is recommended to use two or more methods as complementary.
(8) The damping nonlinearity identification process is very complicated due to the presence of a mixture of different damping mechanisms at the same time. Therefore, in many cases, the theoretical study for the NDI in structures should be followed by experimental work to validate the results.

## Figures and Tables

**Table 1:** Literature survey of nonlinear damping identification methods.

Linearization Methods							
**Method**	Function	DOF	Limitations	Advantages	Applications	Damping Type	Ref.
Linearization method	Predict the response to packaged components from nonlinear systems	MDOF	The transient response is not included.	A powerful tool Able to analyze complex shapes	Packaged Components	Viscous damping	[78]
Output-only system identification technique	Hysteretic damping estimation	SDOF	Needs assumptions. limited to a narrow band response.	Suitable for the random response	MR Dampers.	Hysteretic damping	[79]
Classical equivalent linearization method	Calculation of the self-excited force in bridge tests.	-	-	Accurate	Bridge; SSSM.	Viscous damping	[80]
Equivalent Linearization Approximation	A study of the mechanical nonlinearity of an SSSM system	SDOF	-	Reliable and precise Predict the long-duration free decay response of the SSSM system	Bridge; SSSM.	Viscous damping, quadratic damping, and Coulomb friction damping	[81]
Equivalent Linearization Approximation	Determination of the nonlinearity of the HAPB system	SDOF	-	Reliable and accurate Predict the long-duration free decay responses	A Spring-Suspension System.	Viscous damping	[82]
**Time-Domain Methods**							
**Method**	**Function**	**DOF**	**Limitations**	**Advantages**	**Applications**	**Damping Type**	**Ref.**
Matrix pencil methods, Envelope functions via the Hilbert transform, Log decrementand Half-power bandwidth	Flutter identification in the flight envelope and create design improvements to alleviate unwanted aeroelastic behavior	-	Sensitive to some initial parameters	Robust to noise and capability for handling multi-component signals across a short-time simulation	Aeroelastic Systems	Aeroelastic damping	[85]
FREEVIB method and Nonlinear decrement method	Investigate the validity of the two identification methods to a SDOF fluid elastic system using experimental data.	SDOF	Requires prior knowledge of the system functional form when using the nonlinear decrement method.	Superior predictions Low errors Combined methods provide a powerful tool No prior knowledge required for FREEVIB method	Heat Exchanger Tube Arrays	Nonlinear cubic damping Structural damping Structural viscous damping	[86]
A decrement method	Linear and nonlinear damping parameters are defined in the fluid elastic framework.	SDOF	Limited to SDOF systems	Required one response measurement	A Slightingly Damped System	Linear and cubic damping	[87]
Random decrement signature approach	Damage detection of RC bridge using a nonlinear damping ratio damage index.	SDOF	-	Damage detection without any reference to the intact baseline.	Bridge; RC Structure	-	[88]
A moving rectangle window method	Simultaneous identification of Coulomb friction and the nonlinear damping.	SDOF	-	Accurate and applicable Extendable to MDOF systems	Mechanical Systems	Viscous damping and the Coulomb friction damping	[89]
Transient time-domain methods	Quantification of some unwanted effects on the overall value of the measured damping.	MDOF	-	Minimize the effects of external damping losses	Steel alloys	Air damping	[90]
Galerkin Method and Finite Difference Method	Investigation of the nonlinear dynamic response of a laminated composite plate under blast loads with damping influences.	MDOF	Higher modes are not included in their contribution to the dynamic response	Can be used to study many properties	A hybrid laminated composite plate	Viscous damping	[91]
Hilbert transform	Nonlinearity determination in stiffness and damping properties of vibration systems.	SDOF	The need for very precise data without noise	-Effective and simple to analyze Proper for linear and nonlinear systems Does not require knowledge of system signals or parameters Reduces testing time without reducing data accuracy	A vibration system	-	[92]
**Frequency-Domain Methods**							
**Method**	**Function**	**DOF**	**Limitations**	**Advantages**	**Applications**	**Damping Type**	**Ref.**
Modified half-power bandwidth method	Study the damping identification of nonlinear stiffness of a titanium alloy	SDOF	-	Broad and higher resolution than the half-bandwidth method.	Titanium Alloy	Equivalent viscous damping	[99]
HBNID methodology	Extension of the HBNID to include the MDOF systems	MDOF SDOF	Poor estimate when the model structure is unknown.	Provides very good and accurate results with a known model structure.	Fluid-elastic systems	-	[100]
Frequency domain method	Nonlinear damping identification of a silicon rubber plate	SDOF	-	Does not require adjustment of the dissipation parameters	A rubber plate	Three different damping models	[101]
Frequency domain approach	Study the application of the ideal nonlinear damping characteristics for an engineering system	SDOF	-	Provides insight into vibration isolation and system stability.	Vehicle suspension system; MR dampers	-	[102]
Experimental FRFs	Investigation of the effects of damages on the effective damping of the viscoelastic adhesive joint	-	-	Study linear and nonlinear areas	The adhesive joint of automobile and aircraft	-	[103]
Inverse Wave Method	Estimation of the damping loss factor of a complex structure using a scanning laser vibrometer in two dimensions	MDOF	-	Simple for structural characterization Accurate and reliable for wavenumber and damping loss factor estimation.	Two-dimensional orthotropic structures	-	[104]
Harmonic Balance Method	Study the softening influence for high displacement amplitudes of a nonlinear rubber isolator	SDOF	-	Simple, valid and global method	A rubber isolator in Aerospace, sensors and bio-engineering	-	[105]
Frequency domain methods	Study nonlinear quadratic damping features of a cantilever beam under harmonic base excitation	SDOF	-	Using dimensionless quadratic damping coefficient for generality and comparability to other structures	Cantilever beams	Nonlinear quadratic damping	[106]
**Time-Frequency Methods**							
**Method**	**Function**	**DOF**	**Limitations**	**Advantages**	**Applications**	**Damping Type**	**Ref.**
Hilbert transform and compared with traditional logarithmic decrement technique	Investigate a nonlinear roll damping and restoring moment of a floating production system	SDOF	Including nonlinear terms reduces logarithmic decrement precision. The nonlinear damping coefficient is not precisely quadratic.	Both Hilbert transform and logarithmic decrement are accurate	Ship and offshore	Quadratic damping	[114]
MIMO curve fitting and Hilbert transform technique	Investigation of the RC beam damage detection method using free vibration measurements and nonlinear damping identification	MDOF SDOF	The scope of application is limited due to the difficulty in obtaining free vibration responses	Easy and suitable for manufacturing quality control of RPC members and extendable to detect damages in concrete structures	Damage detection of RC beams	A nonlinear quadratic damping	[115]
Hilbert transform and compared with the RFS method	Study the identification of the nonlinear vibration absorber parameters of rotating machines	SDOF	-	Gives error only about 13% compared with the RFS method	Rotating machines	Cubic stiffness and viscous damping	[116]
Continuous wavelet transforms	Study NDI method using CWT for the rotor-bearing system	MDOF	-	Does not require an analytical solution of the signal	Unbalance of a rotor-bearing system	Quadratic and cubic polynomial type nonlinearities	[117]
Wavelet transform; cross-section procedure and ridge and skeleton of the WT	Estimation of instantaneous frequency, damping, and system envelopes using wavelet transform for a broad range of engineering applications	SDOF	Limited because it cannot give accurate results with high levels of noise	Cross-section procedures give satisfactory results at low levels of noise. Ridge procedure yields accurate results at high levels of noise.	Many engineering applications	A special class of nonlinear damping models characterized by low damping	[118]
Wavelet transform	Investigate a structural damage detection scheme for RC using an instantaneous damping coefficient identification applying a WT	MDOF	-	Easily used in instantaneous identification procedures of frequency and damping from the response of the free vibration	Damage detection of RC	-	[119]
Wavelet transform	Estimation of the effect of mechanical joints on the dynamic behavior of two bolted beams	MDOF	-	-	A simple structure of two beams	Equivalent damping coefficient	[120]
Continuous Wavelet Transform	Study the dynamics of a TLCD focusing on the frequency and nonlinear identification and air pressure characterization	-	-	The quadratic damping model can accurately describe the dissipative behavior	Naval architecture; Vibration absorber	A quadratic damping model	[121]
**Modal Methods**							
**Method**	**Function**	**DOF**	**Limitations**	**Advantages**	**Applications**	**Damping Type**	**Ref.**
Modal methods Transmissibility measured data Numerical simulations	Study the dynamic properties of a metal mesh isolator under various excitation levels to enhance the transmitted vibrations reduction	SDOF	Include some errors in the estimation of damping and the effect of the jump phenomenon	Accurate	Anti-vibration isolators; many engineering applications	Quadratic damping and cubic stiffness	[127]
Resonant Decay Method (RDM)	Identification of the modal matrix element of nonproportional damped systems of a plate with discrete dampers	MDOF	-	This method yields acceptable and accurate modal damping matrices	Plate with discrete dampers	Viscous damping	[128]
Modified RDM	Extraction of the backbone curves of the lightly damped nonlinear systems using a modified RDM	SDOF MDOF	Low accuracy when identifying the amount of damping	Strong ability to achieve an accurate evaluation of damping ratio skeletons and backbone curves.	Civil aircraft	Three different models	[129]
Nonlinear modal analysis technique; a ROM method	The nonlinear modal characteristics were utilized to evaluate the forced and self-excited vibration.	2-DOF MDOF	Only nonlinearities of steady-state problems	Very good agreement with results obtained by conventional approaches.	Mechanical systems; a clamped beam and a turbine bladed disk	Viscous damping, hysteretic damping, and modal damping	[130]
Nonlinear modal analysis; Harmonic Balance method and Shooting method	Estimation of the nonlinear modal parameter of nonconservative nonlinear systems	SDOF MDOF	Limited to the isolated nonlinear modes and low modal damping ratios Restricted to periodic motions	Provides accurate predictions for a broad range of working conditions	Nonconservative systems;	Viscous damping and Friction damping	[131]
Nonlinear modal analysis; Harmonic Balance method and Shooting method and a nonlinear phase resonance method	Identification of nonlinear modal parameters of non-smooth mechanical systems	MDOF	Complex structures with strong nonlinearity are not included.	The numerical method can be applied without requiring any effort to define the nonlinear system.	Mechanical system: Timoshenko beam	A nonlinear modal damping	[132]
Nonlinear modal analysis	Study the extension of nonlinear modal testing by a considerably better accurate damping quantification of Jointed structures such as modern turbine blades	MDOF	-	Requires only one signal response for each vibration level and does not require special equipment. It is efficient, time-saving, and robust against noises. Accurate and applicable to realistic applications.	Jointed structures; modern turbine blades	Modal damping ratio	[133]
Experimental modal analysis	Estimation of nonlinear modal characteristics of a cantilever beam with strong damping nonlinearity	SDOF	-	Accurate at different excitation levels	Jointed structures	Friction damping	[134]
Nonlinear experimental modal analysis	Identification of nonlinear modal parameters of strongly nonlinear systems	MDOF	Restricted to systems have separated modes	Accurate even with very strong nonlinear effects	Jointed structures;	Nonlinear hysteretic modal damping	[135]
**Black-Box Modeling**							
**Method**	**Function**	**DOF**	**Limitations**	**Advantages**	**Applications**	**Damping Type**	**Ref.**
BBM; A neural network-based output error model	Study the black-box estimation of electro-hydraulic semi-active dampers for vehicles	-	-	An accurate model Suitable for a full car simulation	An electro-hydraulic semi-active damper; vehicle suspension	-	[138]
BBM and IBBM based on a fuzzy-neural technique	Study the magneto-rheological fluid dampers using the force-sensor less control technique for vibration control	-	-	A direct method for damper characterization	Control systems; optics, defense, aerospace, automotive	-	[139]
BBM neural networks	Investigation of the efficacy of the method of the neural network for describing the dynamic behavior of an MR damper used in control systems	-	-	Able to predict the responses over a broader range of operating conditions Avoids large sets of data produced throughout the collection process	Civil structures, automotive, aviation, Control	-	[140]
Using fuzzy wavelet neural network (FWNN)	Investigation of a nonlinear identification method based on a fuzzy wavelet neural network for the two-dimensional wing section	Pitch DOF	-	Able to model uncertainty and subsequent parts. High accurate method in numerical investigations.	Two-dimensional wing section	Viscous damping	[141]
**Model Updating Methods**							
**Method**	**Function**	**DOF**	**Limitations**	**Advantages**	**Applications**	**Damping Type**	**Ref.**
Finite element model updating procedure	Damping identification to accurately predict the measured FRFs using finite element updated models of the structural systems	MDOF	-	An accurate method for predicting the complex FRFs. It can be applied to actual applications	Mechanical engineering	Non-proportional viscous damping model	[148]
FRF-based model updating technique	Identification of the structural damping utilizing the FRF-based model updating technique	MDOF	-	Direct and explicit method Provides accurate predictions of FRFs collected from the experiment with all damping levels Can determine the structural damping of the system with closely spaced modes	Mechanical engineering	Structural damping	[149]

## Abbreviations

The following abbreviations are used in this paper:

BBM	Black-box modeling
CFD	Computational fluid dynamics
CHGB	Compliantly damped hybrid gas bearings
CWT	Continuous wavelet transform
EL	Equivalent linearization
ELA	Equivalent linearization approximation
FE	Finite element
FREEVIB	Free vibration analysis
FRFs	Frequency response functions
FWNN	Fuzzy wavelet neural network
HAPB	Hybrid aeroelastic pressure balance
HBNID	Harmonic balance nonlinearity identification
HT	Hilbert transforms
MDOF	Multi-degree of freedom
MIMO	Multi-input multi-output
MR	Magnetorheological
NDI	Nonlinear damping identification
PRC	Prestressed reinforced concrete
RC	Reinforced concrete
RCT	Response-controlled stepped-sine testing
RDM	Resonant decay method
RDT	Random decrement technique
RFS	Restoring force surface
R-K	Runge-Kutta
ROM	Reduced-order model
SDOF	Single-degree of freedom
SSSM	Spring-suspended sectional models
TLCD	Tuned liquid column damper
WT	Wavelet transform
